# Evaluation of Corrosive Properties of Hafnium Nitride Coating Over Titanium Screws: An In Vitro Study

**DOI:** 10.7759/cureus.55456

**Published:** 2024-03-03

**Authors:** Vaishnavi Rajaraman, Padma Ariga, Karthikeyan Ramalingam, Saravanan Sekaran

**Affiliations:** 1 Prosthodontics, Saveetha Dental College and Hospitals, Saveetha Institute of Medical and Technical Sciences, Saveetha University, Chennai, IND; 2 Oral Pathology and Microbiology, Saveetha Dental College and Hospitals, Saveetha Institute of Medical and Technical Sciences, Saveetha University, Chennai, IND

**Keywords:** titanium, uncoated, polarization, impedance, hafnium nitride, corrosion, coated

## Abstract

Background

Varied surface coatings have been studied time and again in medical sciences. Whether general or dental, studying the performance of coatings aims to assess their potential to improve the durability and longevity of titanium implants, thereby advancing implant technology for enhanced patient outcomes. Various analytical techniques are utilized to assess the performance of the coating, providing insights into its effectiveness in preventing corrosion. The findings of this evaluation will contribute to our understanding of corrosion mitigation strategies for titanium implants and pave the way for the development of more durable implant materials. This article aims to evaluate the corrosion resistance of an innovative metal compound coating applied over titanium implants.

Materials and methods

In this study, a total of 20 medical-grade, commercially pure titanium screws were collected. The dimensions of the titanium screws were 2mm x 7mm. Around 10 of these commercially pure titanium screw samples were used as the control group. Hafnium nitride (HfN) (0.1 M) was mixed with 100% ethanol and stirred using a glass rod for about 48 hours. Then 10 of the implant screw samples were immersed in the prepared sol and sintered at 400^o^ C for two hours. The HfN-coated samples were then used as the test group. The corrosion resistance of both groups was tested using electrochemical impedance spectroscopy and potentiodynamic polarization studies. The Nyquist, Bode impedance, and Bode phase angle plots were obtained and studied.

Results

Using the Stern-Geary equation, the corrosion current density was calculated. On analysis, these values indicated that the higher impedance in HfN-coated titanium screws showed higher mean corrosion potential (E_corr_ = -0.452 V) and corrosion current density ( i_corr_ = 0.0354 μA/cm^2^) than the uncoated titanium screws.

Conclusion

It was concluded that the corrosion properties of HfN-coated titanium screws had higher impedance and consequently the highest corrosion resistance. This thereby provides a promising scope for further research of this novel metal coating for use in the biomedical sectors, specifically for dental implants.

## Introduction

Titanium screws are commonly employed in prosthetic surgeries due to their excellent mechanical and biological properties [1,2]. Although titanium is considered the gold standard to date in dental implantology, researchers keep exploring alternative biomaterials. However, concerns persist regarding their susceptibility to corrosion in physiological environments, which can compromise implant stability and long-term performance [3,4]. One such approach involves the application of specialized metal coatings on the implant surface, which can provide a protective barrier against corrosive environments [5,6].

The use of surface coatings to enhance the corrosion resistance of metallic implants has garnered significant interest in biomedical applications. The element, Hafnium, belongs to period 6 in the periodic table, similar to gold standard titanium [7]. In our previous study on rat mandibles, hafnium coating on endosseous implants showed equivalent osseointegration to titanium, the gold standard [8]. Hafnium is similar to titanium in its biocompatibility with bone tissues [5,8]. Hafnium nitride (HfN) coatings have emerged as a promising solution to mitigate corrosion in titanium implants [9,10]. 

HfN could exhibit corrosion resistance properties and can effectively protect the underlying substrate from degradation [11]. Despite the growing interest in HfN coatings, comprehensive evaluations of their surface topography and corrosion resistance on titanium screws remain limited. This material holds significant scope for the development of implant biomaterials that are resistant to corrosion, particularly in orthopedic and dental surgeries [12]. By comprehensively evaluating the surface topography and corrosion resistance of HfN-coated titanium screws, this study aims to advance our understanding of protective coating technologies for biomedical prospects. 

The findings from this investigation may guide the way for the design and optimization of HfN coatings to enhance the durability and reliability of implant materials, ultimately benefiting patient outcomes and healthcare practices. Our investigation aims to elucidate the corrosion behavior of HfN, ultimately contributing to the advancement of implant materials in biomedical applications.

## Materials and methods

Study design and sample preparation

The current study was designed and executed in the Green Lab, Research Cell, in the university set-up of Saveetha Dental College and Hospitals, India, after obtaining approval from the institutional review board for research, with the allocated project number SRB/SDC/UG-1837/23/PROSTHO/010. In this study, a total of 20 medical-grade titanium screws were collected. As this was a pilot study of its kind without a reference study, a sample size of 20 was chosen. Medical-grade titanium implant screws were purchased from G.R. Bioure Surgical System, Pvt. Ltd (Ravali, India). The dimensions of the titanium screws were 2mm x 7mm, in line with those used in previous studies [13]. HfN (0.1 M) from nano Research Elements (Behlolpur, India) was mixed with 100% ethanol and stirred using a glass rod for about 48 hours. Then 10 of the implant screw samples were immersed in the prepared sol and sintered at 400^o^ C for two hours. The coated samples were then used as the test group.

**Figure 1:**
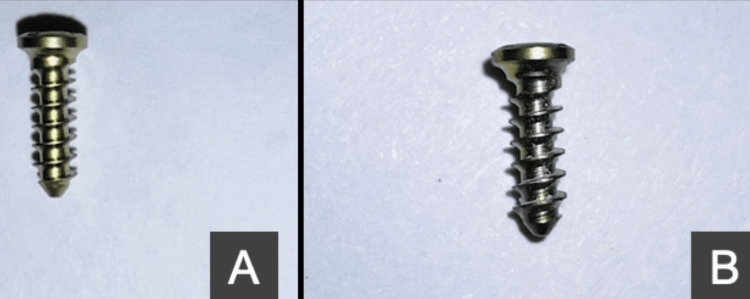
Figure showing a sample of (a) HfN-coated titanium screws; (b) commercially pure titanium screws HfN: hafnium nitride

In total, 10 uncoated commercially pure medical-grade titanium screws were taken as the control group and 10 HfN-coated titanium screws were taken as the test group (Figures 1A-1B).

Electrochemical impedance spectroscopy

A workstation (PGSTAT model 302 N®, Metrohm Autolab B.V., Netherlands) that was electrochemical in nature, guided by NOVA 2.0® software, was used to conduct potentiodynamic polarization tests and electrochemical impedance spectroscopy (EIS) analysis on the samples. It uses a three-electrode electrochemical cell with a platinum foil and saturated calomel electrode (SCE) as the counter and reference electrodes [14]. The sample was employed as a working electrode with a 1 cm^2^ exposed surface area. Simulated body fluid (SBF) solution was used for the corrosion investigations [15]. The sample was submerged for one hour in SBF solution to get a stable open circuit potential (OCP). A 10 mV in the frequency range was applied as a sinusoidal voltage for the EIS investigations.

Potentiodynamic polarization

The potential range used for the potentiodynamic polarization investigations was -1 to 1 V, and the potentials were recorded with respect to SCE at a scan rate of 1 mVs ^-1^ [16,17]. This graphic data was used to determine the corrosion potential (E_corr_) and corrosion current density (i_corr_) was determined using a formula. The Stern-Geary equation was used to get the corrosion current density [18,19].

## Results

**Figure 2:**
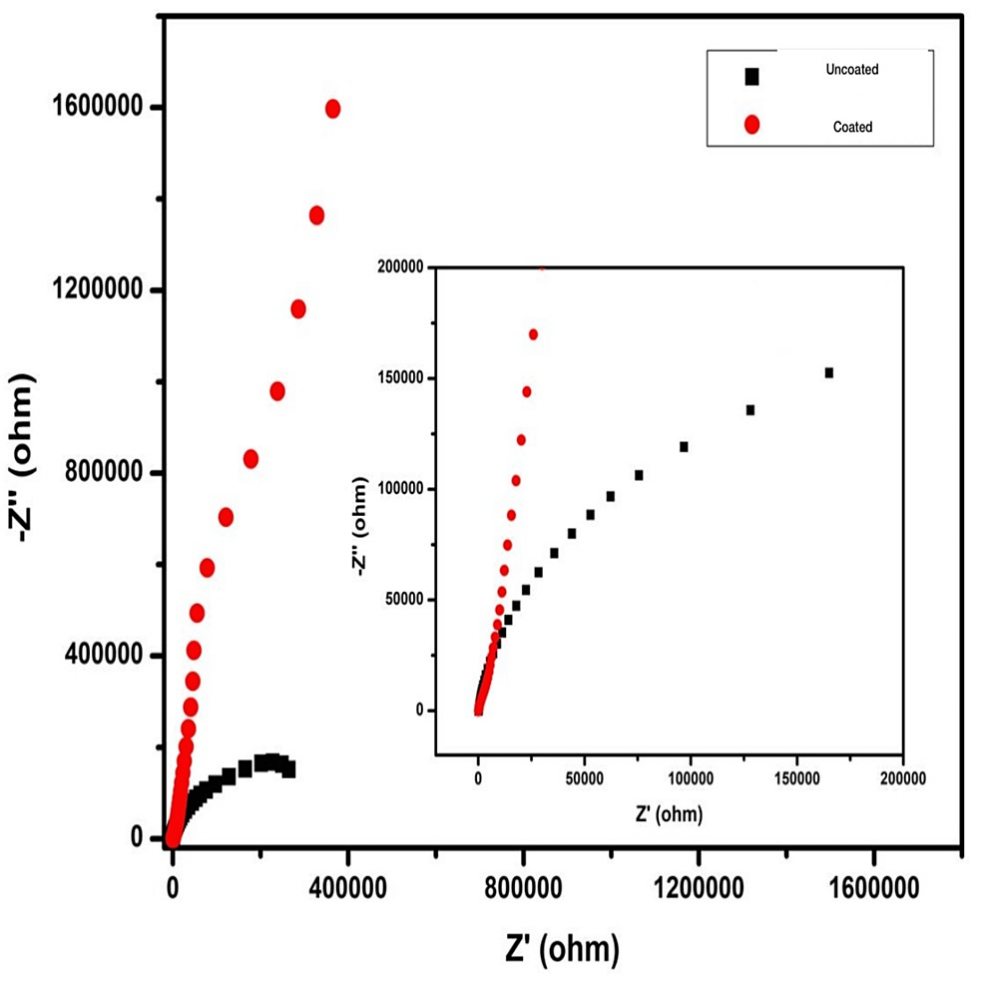
The Nyquist plot showing EIS investigation of the uncoated titanium screws (black) and the HfN-coated titanium screws (red). HfN-coated screws show superior impedance spectra than uncoated titanium. HfN=hafnium nitride

The EIS showed that HfN-coated screws showed superior impedance, Z (ohm) spectra as compared to that of uncoated titanium screws (Figure 2).

**Figure 3:**
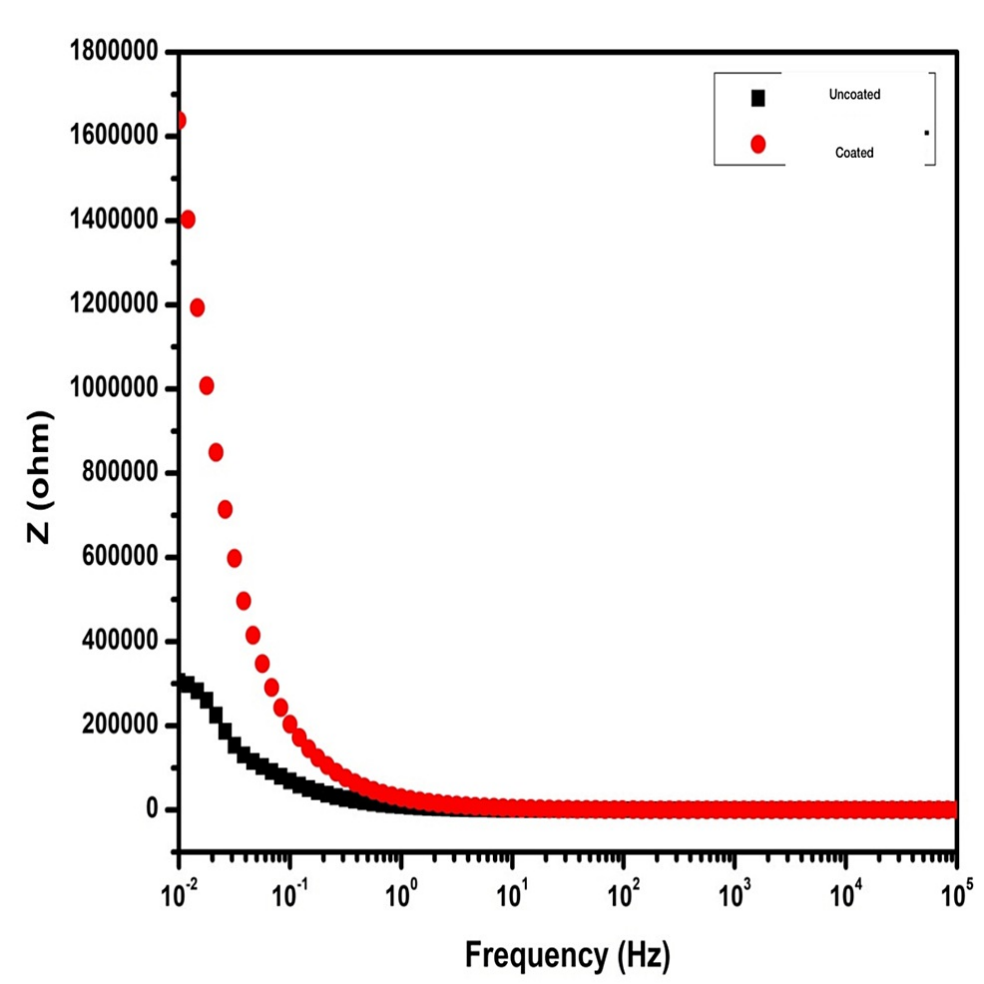
The Bode impedance plot showing frequency (Hz) versus impedance (Z) of the uncoated titanium screws (black) and the HfN-coated titanium screws (red). The HfN-coated screws show superior impedance spectra than uncoated titanium. HfN=hafnium nitride

The Bode impedance plot also showed similar results skewed toward the HfN-coated group (Figure 3).

**Figure 4:**
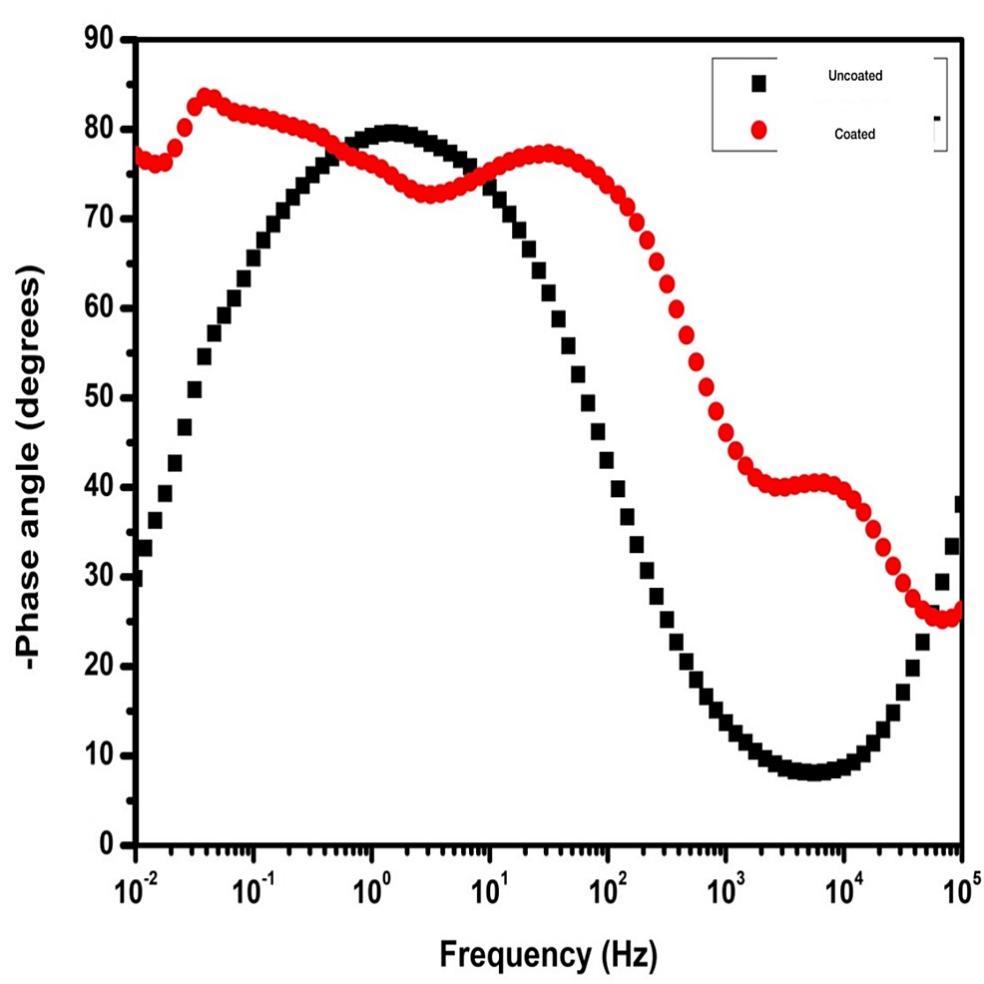
The plot of the Bode phase angle showing frequency (Hz) versus phase angle (degrees) of the uncoated titanium screws (black) and the HfN-coated titanium screws (red). HfN-coated screws show a higher Bode phase angle than uncoated titanium. HfN: hafnium nitride

The Bode phase angle plot showed a higher Bode phase angle for HfN-coated screws than uncoated titanium (Figure 4).

**Figure 5:**
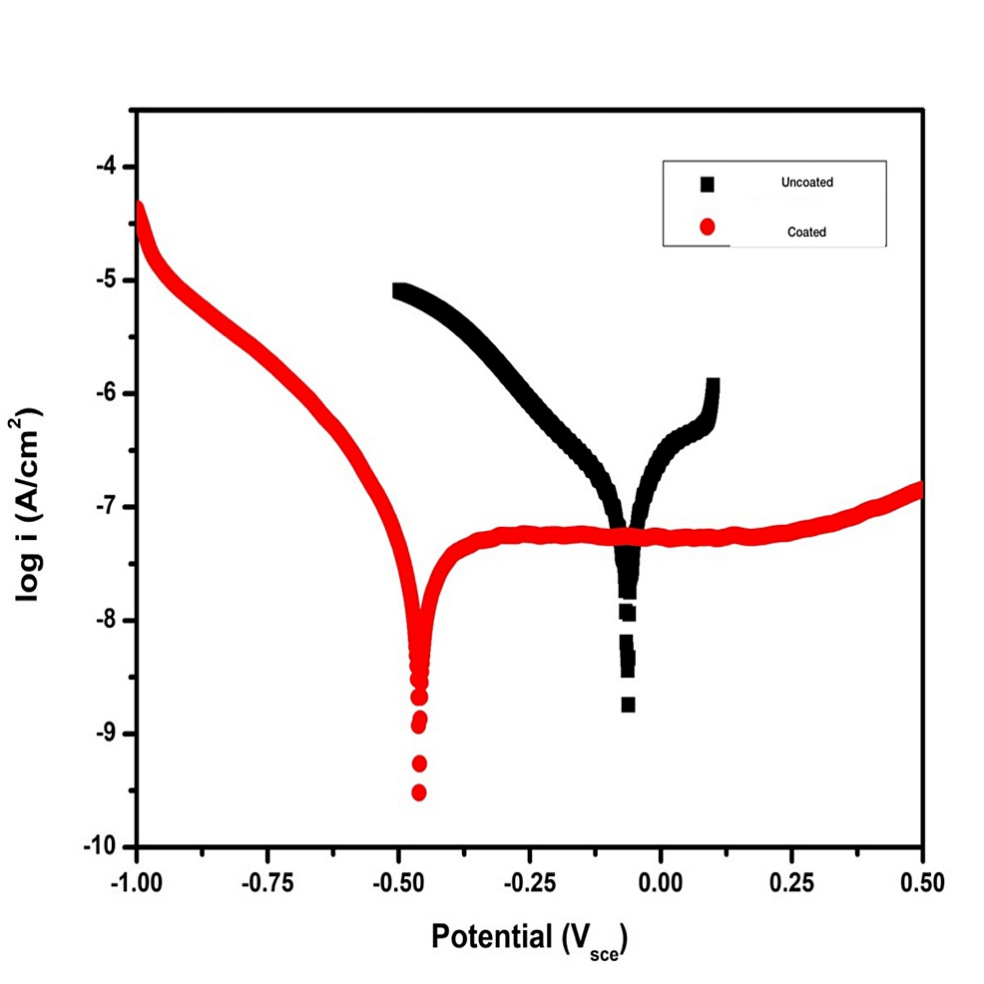
The graph showing the corrosion potential (V SCE) versus log i. (A/cm2) for uncoated titanium screws (black line) and HfN-coated titanium screws (red line). SCE=saturated calomel electrode; HfN=hafnium nitride

The potentiodynamic polarization study showed mean corrosion potential, E_corr_ = -0.088 V for uncoated titanium screws and E_corr_ = -0.452 V for HfN-coated titanium screws. This data was obtained using the graph plotted for corrosion potential (V SCE)versus log i (A/cm2) (Figure 5). 

The corrosion current density for uncoated titanium screws was i_corr_ = 0.1527 μA/cm2 and for HfN-coated titanium screws was i_corr_ = 0.0354 μA/cm2, which was calculated using the Stern-Geary equation (Table 1).

**Table 1:** Table showing the anodic Tafel slope (βa), cathodic Tafel slope (-βc), mean corrosion potential (Ecorr), corrosion current density (icorr) HfN= hafnium nitride; i_corr_ = mean corrosion current density; βa = anodic Tafel slope; βc = cathodic Tafel slope; Rp = polarization resistance

Groups	E_corr_ (V_SCE_)	i _corr_ (μA/cm^2^)	βa	-βc
Uncoated	-0.088	0.1527	3.1522	4.814
HfN-coated	-0.452	0.0354	8.128	2.100

## Discussion

In this study, we have evaluated the surface coating of HfN over titanium screws. The corrosion properties of this compound were evaluated using EIS investigation which suggested that the HfN-coated group had higher impedance and consequently the highest corrosion resistance with mean corrosion potential, E_corr_ = -0.452 V, and corrosion current i_corr_ = 0.0354 μA/cm2. The Nyquist, Bode impedance, and Bode phase angle plots all provided similar findings. From the results, we can conclude a positive corrosion resistance by HfN coating. 

The development of corrosion-resistant titanium implants is of paramount importance in the medical field. Corrosion poses a significant challenge in medical implants, as it can compromise the structural integrity of the implant and lead to adverse biological reactions in the host organism [20,21]. By addressing the challenges associated with corrosion, researchers and healthcare professionals aim to enhance patient safety, improve implant durability, maintain mechanical stability, preserve aesthetics, and potentially reduce the risk of infections. The evaluation of resistance to corrosion is a critical aspect in assessing the performance and longevity of innovative metal coatings applied to titanium implants. In this study, the focus is on understanding the effectiveness of HfN coating designed to enhance the corrosion resistance of titanium implants.

The evaluation process involves a comprehensive set of tests and analyses. Researchers have conducted tests aimed at replicating the corrosive effects of bodily fluids [22], temperature variations [23], and mechanical stresses [21,23] to which the implants may be subjected over time. However, the literature lacks enough evidence to substantiate the ideal method for evaluating corrosion resistance. In previous studies, the potentiodynamic polarization technique has been widely used to evaluate the corrosion resistance of biomaterials [22]. In this way, it is easy to measure the polarization curves of metallic materials. Using the existing evidence, the most commonly used methods of analysis of corrosion resistance for all biological environment-related studies were narrowed down. These were the tests used in this current research. Testing techniques such as potentiodynamic polarization and EIS are employed in current research to simulate the diverse environmental conditions that implants may encounter within the human body [24-26]. 

Limitations

Delving into the biocompatibility of the coating and evaluating its impact on cell viability, adhesion, and proliferation were not considered in this research. A major limitation of this study also includes the fact that the coating surface morphology and adherence were not taken into account or evaluated. Furthermore, the biological aspect of corrosion resistance could be explored for future studies. This is essential to ensure that the corrosion-resistant coating not only performs well in simulated environmental conditions but also maintains a favorable interaction with the surrounding biological tissues.

## Conclusions

In conclusion, the corrosion properties of HfN-coated titanium screws had higher impedance and consequently the highest corrosion resistance when compared to uncoated titanium screws. The evaluation of corrosion resistance for the HfN coating over titanium implants is a multifaceted process that combines electrochemical, material, and biological analyses. In this study, we have concentrated only on the corrosive properties of the novel coating. The goal is to provide a comprehensive understanding of the coating's performance under realistic conditions, ultimately contributing to the development of more durable and biocompatible implants for enhanced patient outcomes.
